# Statistical analyses and quality of individual participant data network meta-analyses were suboptimal: a cross-sectional study

**DOI:** 10.1186/s12916-020-01591-0

**Published:** 2020-06-01

**Authors:** Ya Gao, Shuzhen Shi, Muyang Li, Xinyue Luo, Ming Liu, Kelu Yang, Junhua Zhang, Fujian Song, Jinhui Tian

**Affiliations:** 1 Evidence-Based Medicine Center, School of Basic Medical Sciences, Lanzhou University, No.199, Donggang West Road, Lanzhou City, 730000 Gansu Province China; 2 The Second Clinical Medical College of Lanzhou University, Lanzhou, China; 3 Evidence-Based Nursing Center, School of Nursing, Lanzhou University, Lanzhou, China; 4 Evidence-Based Medicine Center, Tianjin University of Traditional Chinese Medicine, Tianjin, China; 5 Public Health and Health Services Research, Norwich Medical School, University of East Anglia, Norwich, NR4 7TJ UK; 6 Key Laboratory of Evidence-Based Medicine and Knowledge Translation of Gansu Province, Lanzhou, China

**Keywords:** Network meta-analysis, Individual participant data, Statistical analysis, Methodological quality, Reporting quality

## Abstract

**Background:**

Network meta-analyses using individual participant data (IPD-NMAs) have been increasingly used to compare the effects of multiple interventions. Although there have been many studies on statistical methods for IPD-NMAs, it is unclear whether there are statistical defects in published IPD-NMAs and whether the reporting of statistical analyses has improved. This study aimed to investigate statistical methods used and assess the reporting and methodological quality of IPD-NMAs.

**Methods:**

We searched four bibliographic databases to identify published IPD-NMAs. The methodological quality was assessed using AMSTAR-2 and reporting quality assessed based on PRISMA-IPD and PRISMA-NMA. We performed stratified analyses and correlation analyses to explore the factors that might affect quality.

**Results:**

We identified 21 IPD-NMAs. Only 23.8% of the included IPD-NMAs reported statistical techniques used for missing participant data, 42.9% assessed the consistency, and none assessed the transitivity. None of the included IPD-NMAs reported sources of funding for trials included, only 9.5% stated pre-registration of protocols, and 28.6% assessed the risk of bias in individual studies. For reporting quality, compliance rates were lower than 50.0% for more than half of the items. Less than 15.0% of the IPD-NMAs reported data integrity, presented the network geometry, or clarified risk of bias across studies. IPD-NMAs with statistical or epidemiological authors often better assessed the inconsistency (*P* = 0.017). IPD-NMAs with a priori protocol were associated with higher reporting quality in terms of search (*P* = 0.046), data collection process (*P* = 0.031), and syntheses of results (*P* = 0.006).

**Conclusions:**

The reporting of statistical methods and compliance rates of methodological and reporting items of IPD-NMAs were suboptimal. Authors of future IPD-NMAs should address the identified flaws and strictly adhere to methodological and reporting guidelines.

## Background

Meta-analysis based on individual participant data (IPD) from randomized controlled trials (RCTs) [1, 2] is considered the “gold standard” of meta-analyses. The use of IPD in meta-analysis offers more flexibility in the investigation of patient-level moderators [3], allows the standardization of outcome definitions and analyses across studies [4, 5], and helps improve the quantity and quality of data [6–8]. Network meta-analysis (NMA), also known as mixed treatment comparison or multiple treatments comparison meta-analysis, is a statistical method to directly and indirectly compare the effects of two or more treatments and allows ranking of different treatments [9–12]. Although NMAs usually include aggregate data from RCTs [13], there are many advantages to incorporating IPD into NMAs, including more appropriate investigation of heterogeneity or inconsistency by using advanced modeling strategies to explore subject-level covariates [14–16], and identifying interactions of patient-level effect modifiers [17–19].

Previous studies have evaluated the statistical methods, methodological and reporting characteristics of IPD meta-analyses [2, 20]. A comprehensive scoping review found that indirect comparisons using IPD often failed to report key statistical methods [3], which may lead to biased conclusions. Several studies also assessed the methodological quality and the conduct of published NMAs [21–25]. But no studies have focused solely on the reporting and methodological quality of IPD-NMAs. Researchers have recognized that the use of IPD in NMAs may yield the most trustworthy evidence to inform clinical decision-making [26]. However, when IPD-NMAs have methodological flaws, they could threaten the validity of the results and thus mislead clinical decision-making [16, 27]. Although there have been many studies on statistical methods for IPD-NMAs [26, 28, 29], it is unclear whether there are statistical defects in published IPD-NMAs and whether the reporting of statistical analyses has improved.

The objectives of this study were to explore the general characteristics, statistical analysis methods used, reporting quality, and methodological quality of IPD-NMAs and to identify study-level variables that were associated with the methodological and reporting quality.

## Methods

### Literature search

A comprehensive literature search was conducted on PubMed, Embase.com, Cochrane Library, and Web of Science using the following items: “network meta-analysis”, “mixed treatment comparison meta-analysis”, “mixed treatment meta-analysis”, “multiple treatment comparison meta-analysis”, “multiple treatment meta-analysis”, “indirect comparison”, “individual patient”, “individual participant”, and “patient level”. The detailed search strategy of PubMed is presented in Additional file 1: Appendix Word 1. We performed the initial search on December 13, 2018, and updated the search on June 3, 2019. We also manually retrieved references of included IPD-NMAs and relevant reviews.

### Eligibility criteria

Because NMAs using IPD from RCTs provide the most valid results, we included IPD-NMAs of RCTs that evaluated the clinical effects of three or more interventions for patients in any clinical conditions. There were no restrictions on publication year and language.

Studies including the following were excluded: (1) NMAs did not incorporate IPD; (2) pairwise meta-analyses of IPD; (3) IPD-NMAs did not focus on health care interventions such as etiology and diagnosis; (4) studies that only applied simulated treatment comparison [30], adjusted indirect comparisons [31], or matching adjusted indirect comparisons (MAICs) [32] failing to preserve within-study randomization; and (5) methodological studies, review protocols, abstracts, conference proceedings, and letters to editors.

### Study selection

We used EndNote X8 (Thomson Reuters (Scientific) LLC Philadelphia, PA, USA) to manage the retrieved records. Two review authors (Y.G. and S.Z.S.) independently reviewed titles and abstracts identified through the electronic search. Full reports of any potentially relevant papers were retrieved for further assessment of eligibility. If an IPD-NMA has been updated, we would only include the latest version. Differences of opinion were settled by consensus or referral to a third review author (F.J.S. or J.H.T.).

### Data extraction

We developed a data extraction form using Microsoft Excel 2016 (Microsoft Corp, Redmond, WA, www.microsoft.com) to abstract data on general characteristics and statistical analysis methods used, including publication year, first author, country of corresponding authors, number of authors, journal name, whether a statistician or epidemiologist (based on the author’s current academic position) was involved, whether IPD-NMAs had a priori protocol, funding source (industry, non-industry, unfunded, or not reported), the topic of interest, number of trials included, number of participants included, number of interventions included, format of data included in analysis (individual participant data, aggregate data), whether used Bayesian method or Frequentist method, 1-stage or 2-stage process, statistical techniques used for missing data, methods used to assess heterogeneity, and methods used to assess consistency and transitivity. We piloted the data extraction on a random sample of five included studies and achieved consistency in data item interpretations. Then, four trained authors (Y.G., S.Z.S., M.Y.L., and X.Y.L.) abstracted data from the included IPD-NMAs, and another two reviewers (J.H.Z. and J.H.T.) checked the data. Disagreements were resolved by discussion.

### Reporting and methodological quality assessment

The reporting quality of the included IPD-NMAs was evaluated according to the Preferred Reporting Items for Systematic Review and Meta-Analyses of individual participant data (PRISMA-IPD) statement, which is a checklist of 31 items aimed to improve the completeness and transparency of reporting of systematic reviews (SRs) and meta-analyses of individual participant data [1]. To identify the important information that should be reported in a network meta-analysis, we also applied five supplemental items of Preferred Reporting Items for Systematic Reviews and Meta-analyses extension (PRISMA-NMA) statement (S1. Geometry of the network, S2. Assessment of inconsistency, S3. Presentation of network structure, S4. Summary of network geometry, S5. Exploration for inconsistency) [33]. Each item was rated with “yes” (total compliance), “partial” (partial compliance), or “no” (noncompliance) [24, 34].

We used the Assessment of Multiple Systematic Reviews-2 (AMSTAR-2) tool to assess the methodological quality of the included IPD-NMAs [35]. This tool assesses the methodological quality of SRs across 16 domains, among which seven are critical [35]. Each domain can be rated as “yes” (item fully addressed), “no” (item not addressed), or “partial yes” (item not fully addressed). According to the critical domains, the overall confidence of the quality of each review can be classified as high, moderate, low, or critically low [35]. The quality assessment was conducted by one reviewer (Y.G., S.Z.S., M.Y.L., and X.Y.L.) and verified by another. Possible disagreements were resolved by consensus or with the consultation of a third party (F.J.S. or J.H.T.).

### Data analysis

We applied frequency and percentage to present categorical variables and median and interquartile range (IQR) to present continuous variables. For individual items of reporting and methodological quality, the compliance rate was computed with the number of items acquired “yes” and the total number of the included IPD-NMAs. Then, we created Radar maps and bubble plots using Microsoft Excel 2016 (Microsoft Corp, Redmond, WA, www.microsoft.com) to present the compliance rates. We classified the included IPD-NMAs according to the following characteristics: with or without a statistician or epidemiologist, with or without a priori protocol, non-industry or industry funding, using a Bayesian or a Frequentist method, and using the 1-stage process or the 2-stage process. We then performed Fisher’s exact test to compare the compliance of each PRISMA-IPD, PRISMA-NMA, and AMSTAR-2 item by the above characteristics. The analyses were conducted in Stata (13.0; Stata Corporation, College Station, Texas, USA), and the statistical level of significance was set at *P* < 0.05.

To investigate whether the reporting quality was associated with the methodological quality of IPD-NMAs, we also performed the correlation analysis. We computed the number of items acquired “yes” of PRISMA-IPD and AMSTAR-2 for each IPD-NMA. Then, we conducted the Shapiro-Wilk test and created the Q-Q normal map to evaluate the normality of numbers of items acquired “yes” of PRISMA-IPD and AMSTAR-2 [22, 24], and results indicated that they were normally distributed (Additional file 1: Appendix Word 2). Therefore, we performed the Pearson correlation analysis using Stata (13.0; Stata Corporation, College Station, Texas, USA) to explore the relationship between fully reported PRISMA-IPD items and AMSTAR-2 items.

## Results

### Literature search

**Fig. 1:**
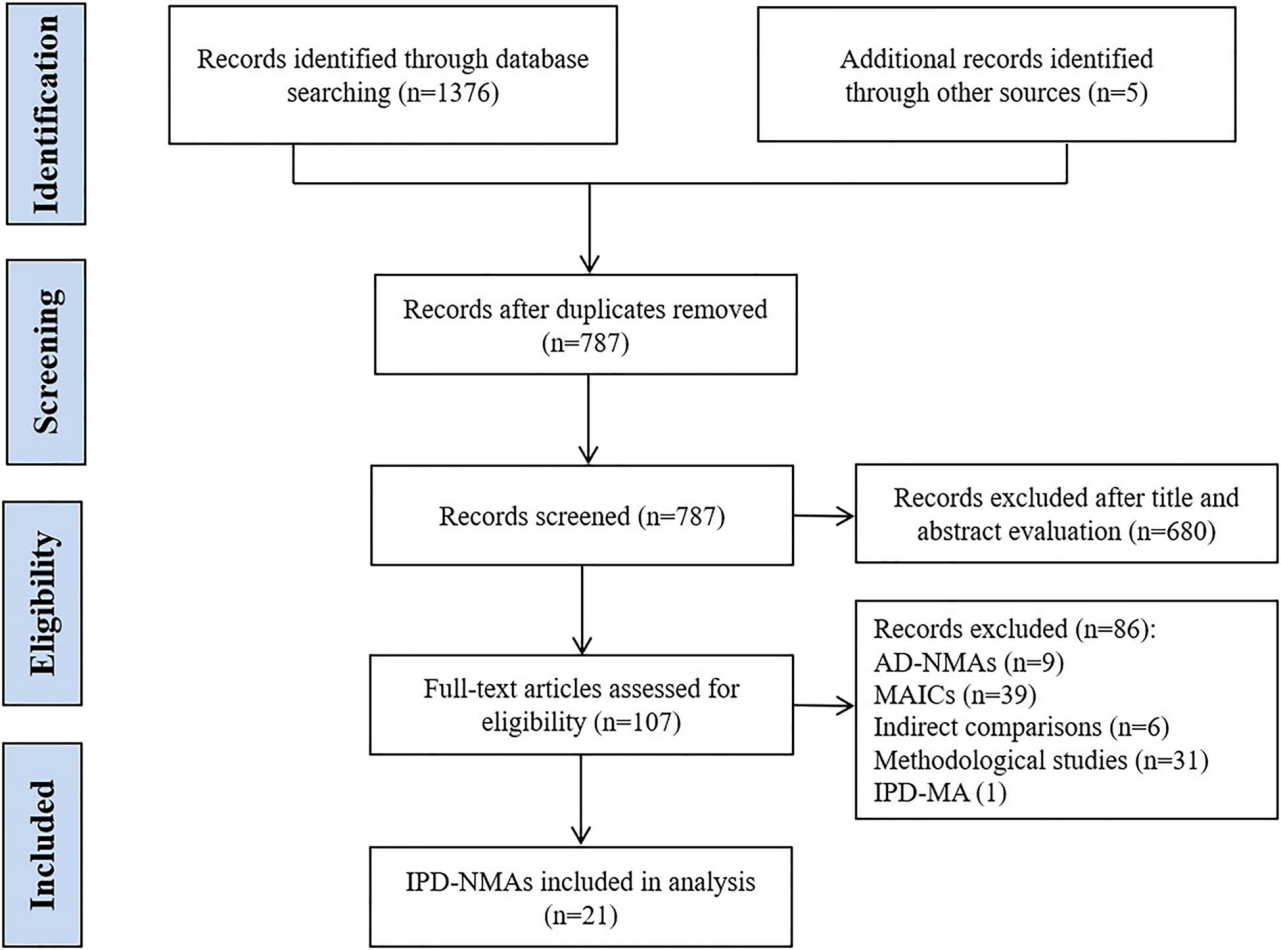
The flowchart of the screening process. AD-NMAs, aggregate data network meta-analyses; MAICs, matching adjusted indirect comparisons; IPD-MAs, individual patient data meta-analyses; IPD-NMAs, individual patient data network meta-analyses

The search of electronic databases yielded 1376 records, and we identified five additional records through manually checking the references of relevant reviews. After removing duplicates and screening titles and abstracts, we identified 107 reports for the full-text assessment. We further excluded 9 aggregate NMAs, 39 MAICs, 6 indirect comparisons, 31 methodological studies, and 1 IPD meta-analysis. Eventually, there were 21 IPD-NMAs that met our eligibility criteria (Fig. 1). A list of the included IPD-NMAs is shown in Additional file 1: Appendix Word 3.

### General characteristics of included IPD-NMAs

**Fig. 2:**
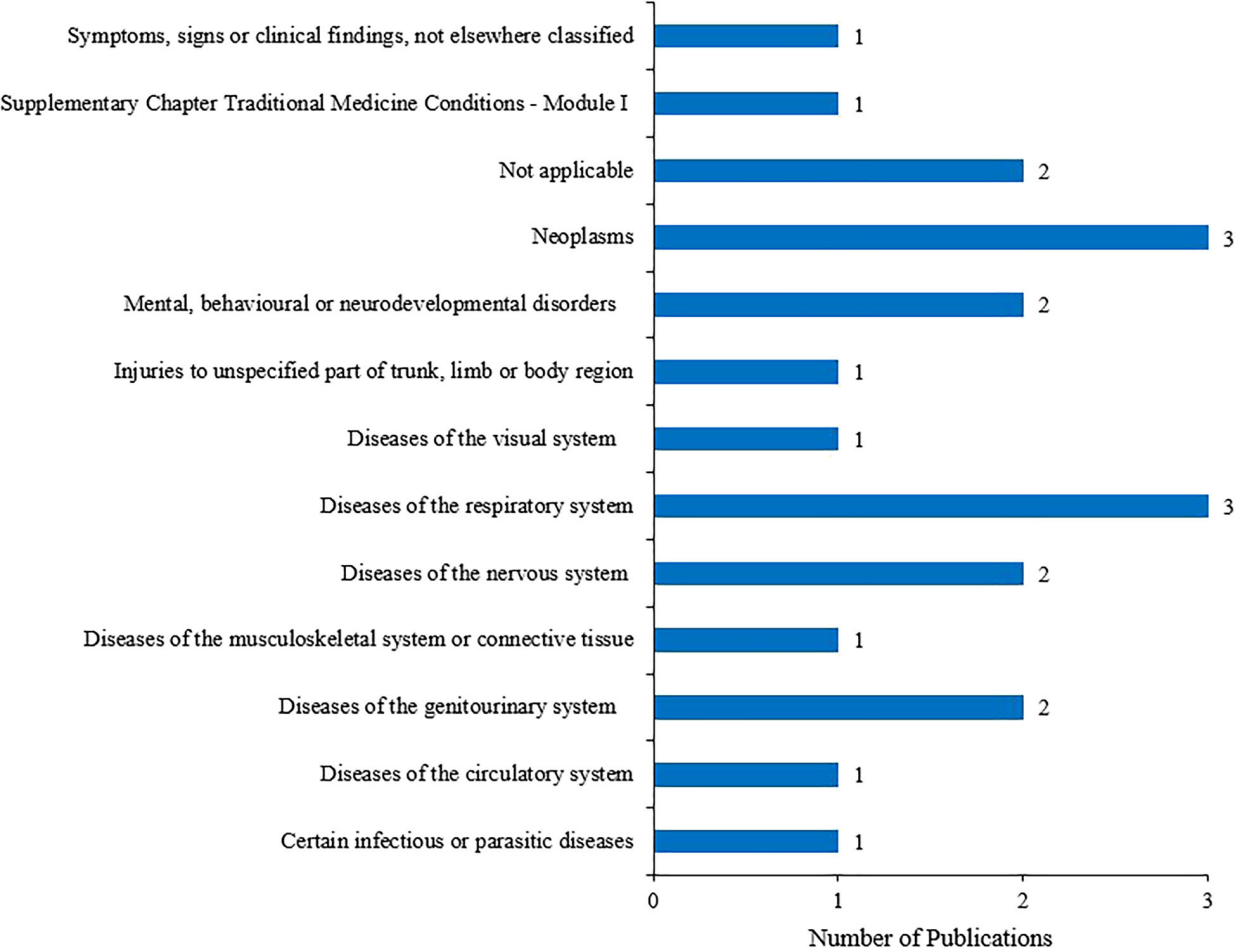
Disease categories of included IPD-NMAs (according to ICD-11). Not applicable if the topic of included SRs does not focus on diseases, such as the dual antiplatelet therapy after drug-eluting stent implantation

The first IPD-NMA was published in 2007, and the remaining were all published since 2010. The USA (9, 42.9%) published the largest number of IPD-NMAs, followed by the UK (7, 33.3%) and France (3, 14.3%). A wide range of different diseases was studied in the included IPD-NMAs (Fig. 2). All the 21 IPD-NMAs were conducted by four or more authors, including eight IPD-NMAs that involved 11 or more authors. Seven (33.3%) IPD-NMAs had statistical or epidemiological authors and 6 (28.6%) IPD-NMAs had a priori protocol. These IPD-NMAs included a median of 19 RCTs, a median of 7110 participants, and evaluated a median of six interventions. The details of the general characteristics are presented in Table 1.

**Table 1:** Characteristics of included IPD-NMAs

Items	Frequency	Proportion (%)
Publication year		
2007	1	4.8
2010	1	4.8
2011	1	4.8
2012	5	23.8
2013	1	4.8
2014	4	19.0
2015	2	9.5
2017	3	14.3
2018	2	9.5
2019	1	4.8
Country of the correspondence author		
USA	9	42.9
UK	7	33.3
France	3	14.3
Cameroon	1	4.8
Netherland	1	4.8
Journal impact factor		
0.0 to 3.0	6	28.6
3.1 to 6.0	5	23.8
6.1 to 15.0	2	9.5
> 15.0	6	28.6
Non-SCI	2	9.5
Number of authors		
1 to 3 authors	0	0
4 to 6 authors	8	38.1
7 to 10 authors	5	23.8
11 or more authors	8	38.1
With statistician or epidemiologist	7	33.3
Authors from 2 or more countries	15	71.4
With a priori protocol	6	28.6
Format of data		
IPD only	11	52.4
IPD + AD	10	47.6
Number of RCTs included: median (IQR)	19 (9, 26)	
Number of samples included: median (IQR)	7110 (4906.5, 14261)	
Number of interventions included: median (IQR)	6 (4,7)	
Funding sources		
Industry	6	28.6
Non-industry	11	52.4
Industry + non-industry	3	14.3
Unfunded	1	4.8

*SCI* science citation index, *IPD* individual participant data, *AD* aggregate data, *IQR* interquartile range

### IPD identification process of included IPD-NMAs

IPD were obtained by forming collaborative research groups in 12 (57.1%) of the included IPD-NMAs, and the collection of IPD was by contacting authors of relevant studies identified through SRs in 7 (33.3%) IPD-NMAs. Three IPD-NMAs reported the proportion of contacted authors who provided IPD, which ranged from 46.8% to 80.0%. IPD-NMAs that identified relevant trials through SRs conducted the literature search, and the commonly used databases were PubMed/MEDLINE, Cochrane Library, and ClinicalTrials.gov (Table 2).

**Table 2:** IPD identification process of included IPD-NMAs

Items	Frequency	Proportion (%)
Methods used to identify IPD eligible studies (*n* = 21)		
Collaborative group^a^	12	57.1
Systematic review and contacting authors	7	33.3
Other methods^b^	2	9.5
Did the authors obtain IPD from all studies or just a subset? (*n* = 21)		
All studies	7	33.3
Not reported	14	66.7
IPD-NMAs that identified IPD through systematic reviews (*n* = 7)		
Proportion of contacted authors provided IPD		
46.8%	1	14.3
70.0%	1	14.3
80.0%	1	14.3
Not reported	4	57.1
Whether a literature search was conducted? (yes)	7	100.0
Number of databases searched		
2 to 5	3	42.9
6 to 9	3	42.9
14	1	14.3
Name of database		
PubMed/MEDLINE	6	85.7
Cochrane Library	6	85.7
ClinicalTrials.gov	6	85.7
EMBASE	4	57.1
Web of Science	2	28.6
ICRTP	2	28.6
Reported the year of retrieval of databases	6	85.7
Presented search strategy	3	42.9
Online supplement	1	14.3
Manuscript	1	14.3
Previous published study	1	14.3

*IPD* individual participant data, *IPD-NMAs* individual participant data network meta-analyses, *ICRTP* World Health Organization International Clinical Trials Registry Platform

^a^IPD-NMAs project team included authors of IPD studies

^b^Other methods mean obtaining IPD from the Yale Open Data Access Project and a previous meta-analysis

### Reporting of statistical analyses of included IPD-NMAs

Table 3 shows the methods of statistical analyses used in the included IPD-NMAs. Fourteen (66.7%) IPD-NMAs used the 1-stage process, and 7 (33.3%) applied the 2-stage process. Twelve (57.1%) IPD-NMAs used a Bayesian method, of which 11 synthesized data using the 1-stage process, and nine (42.9%) used a Frequentist method, of which 6 adopted the 2-stage process. Of the 11 IPD-NMAs included only IPD, 6 applied the Bayesian method, and of the 10 IPD-NMAs incorporated both IPD and aggregate data, 4 applied the Frequentist method. None of the IPD-NMAs clarified the detailed method used for combining IPD with aggregate data. Of the 12 Bayesian IPD-NMAs, all assessed the fit of the model used in data analysis and deviance information criterion (DIC) was the most commonly used method, four did not report the information on the prior distribution, five used the noninformative prior, one used the informative prior, and two adopted the noninformative prior and informative prior. Majority (76.2%) of the IPD-NMAs simply ignored the missing data, three studies adopted the last observation carried forward (LOCF) to handle the missing data, one used the available case analysis (ACA) method, and one used the Markov chain Monte Carlo (MCMC) multiple imputations method. Heterogeneity was assessed in 15 (71.4%) IPD-NMAs, and subgroup analysis and sensitivity analysis were the commonly used methods to explore the sources of heterogeneity. However, only nine IPD-NMAs evaluated the consistency between direct and indirect evidence and none assessed the transitivity.

**Table 3:** Reporting information of statistical analyses of included IPD-NMAs

Items	Frequency	Proportion (%)
Fixed- or random-effects?		
Fixed-effects	6	28.6
Random-effects	6	28.6
Fixed- and random-effects	6	28.6
Not reported	3	14.3
Bayesian or Frequentist method?		
Bayesian	12	57.1
Frequentist	9	42.9
1-stage or 2-stage process?		
1-stage	14	66.7
2-stage	7	33.3
How was the model fit assessed?		
Deviance information criterion	8	38.1
Deviance information criterion + residual deviance	2	9.5
Not reported	2	9.5
Not applicable	9	42.9
Were the prior distributions reported?		
Yes	8	38.1
Noninformative prior^a^	5	23.8
Informative prior^a^	1	4.8
Noninformative prior + informative prior	2	9.5
Not reported	4	19
Not applicable	9	42.9
Was the convergence assessed?		
Yes	8	38.1
Gelman-Rubin statistic	4	19
Visual plot inspection	1	4.8
Gelman-Rubin statistic + visual plot inspection	3	14.3
Not reported	4	19
Not applicable	9	42.9
Statistical techniques used for missing participant data		
LOCF	3	14.3
ACA	1	4.8
MCMC multiple imputations	1	4.8
Not reported	16	76.2
Was the heterogeneity assessed?		
Yes	15	71.4
Not reported	6	28.6
Was the consistency assessed?		
Yes	9	42.9
Loop-specific approach	2	9.5
Node-splitting	2	9.5
Lu and Ades	1	4.8
Lumley	1	4.8
Informal approaches^b^	3	14.3
Not reported	12	57.1
Was the transitivity assessed? (yes)	0	0
Subgroup analysis conducted? (yes)	10	47.6
Sensitivity analysis conducted? (yes)	16	76.2
Meta-regression analysis conducted? (yes)	8	38.1
With GRADE used	2	9.5
Software used		
WinBUGS/OpenBUGS	7	33.3
R	3	14.3
WinBUGS + R	3	14.3
WinBUGS + Stata	1	4.8
SAS + Review Manager^c^	2	9.5
SAS + Stata	1	4.8
R + Stata	1	4.8
Not reported	3	14.3

*IPD-NMAs* individual participant data network meta-analyses, *LOCF* last observation carried forward, *ACA* available case analysis, *MCMC* Markov chain Monte Carlo, *GRADE* Grading of Recommendations Assessment, Development and Evaluation

^a^Priors are based on the detailed methods of prior distribution reported in IPD-NMAs

^b^Informal approaches are the comparison of IPD-NMA with meta-regression IPD-NMA results, comparison of IPD-NMA with aggregate data NMA results, and comparison of NMA results with pairwise meta-analyses results

^c^Review Manager was used for pairwise meta-analyses

### Reporting quality of included IPD-NMAs

**Fig. 3:**
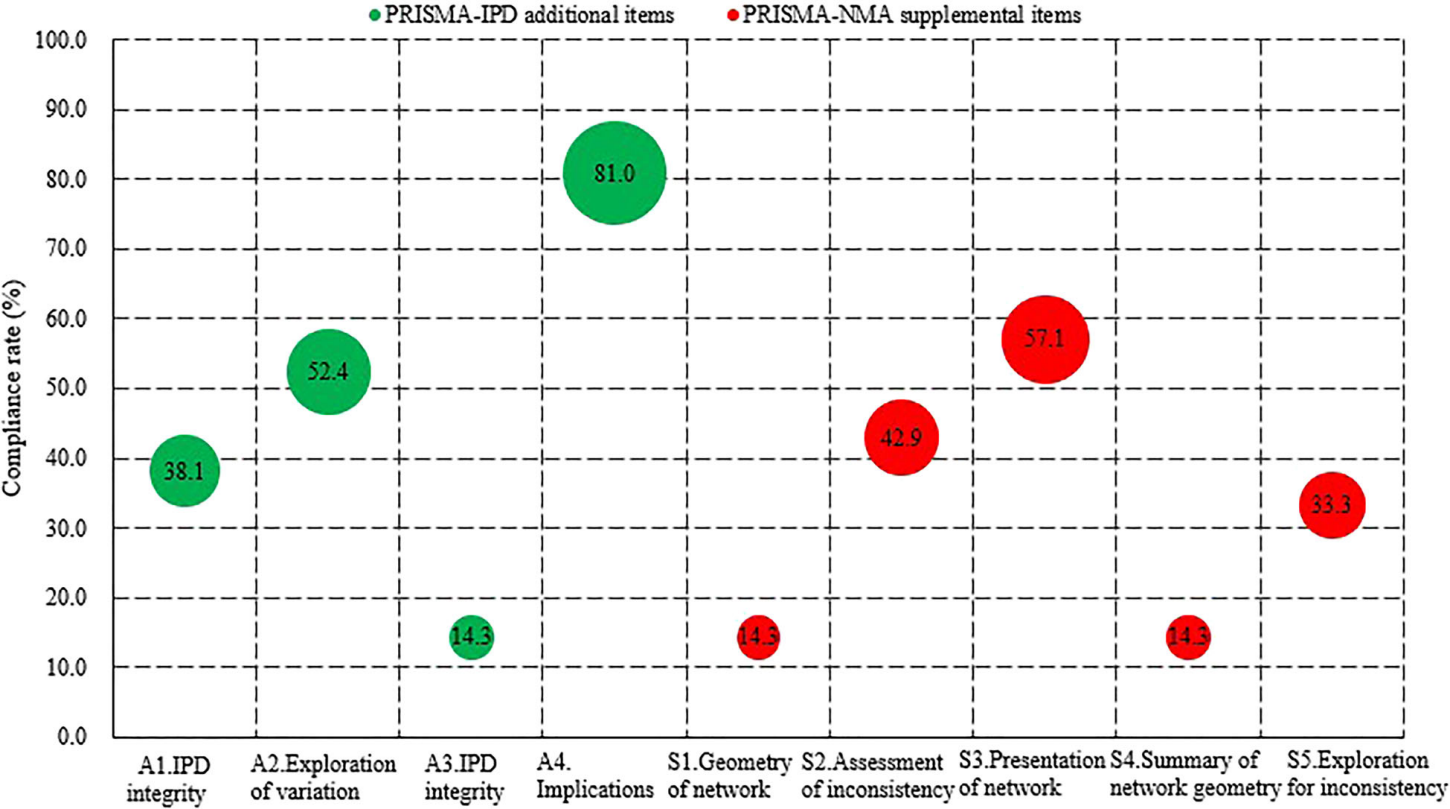
The full compliance rate of each PRISMA-IPD additional item and PRISMA-NMA supplemental item

In terms of the four PRISMA-IPD additional items, the rate of full compliance was 81.0%, 52.4%, 38.1%, and 14.3%, respectively, for implication, exploration of variation, clarification of the IPD integrity in the “Methods” section, and present it in the “Results” section. Regarding the five PRISMA-NMA supplemental items, one item (S3. Presentation of network structure) was fully reported in 57.1% of the IPD-NMAs. However, only 14.3% of the IPD-NMAs described the geometry of network in the “Methods” section and summarized the network geometry in the “Results” section (Fig. 3). Of the remaining 27 items of PRISMA-IPD, the compliance rates of 8 items were higher than 75.0%, and two items (item 3: Rationale, item 26: Conclusions) obtained compliance rates of 100.0%. However, 11 items were not reported in more than 60.0% IPD-NMAs, and 2 items (item 5: Protocol and registration, item 15: Risk of bias across studies) were only presented in 9.5% of the IPD-NMAs (Additional file 2: Appendix Fig. 1).

IPD-NMAs with a statistician or epidemiologist had a significantly higher rate in reporting exploration for inconsistency (71.4% vs. 14.3%) than other IPD-NMAs. A priori protocol was significantly associated with reporting quality in terms of “search” (88.3% vs. 26.7%), “data collection process” (66.7% vs. 13.3%), and “results of syntheses” (83.3% vs. 13.3%). Except for a significant association between the 1-stage process and “data items,” no statistically significant differences were observed between the reporting quality and other factors, including industry funding or not, and Bayesian or Frequentist method (Additional file 1: Appendix Table 1). Improvements were observed in the overall reporting quality (compliance rates of sixteen PRISMA-IPD items and five PRISMA-NMA supplemental items have increased) after the publication of PRISMA-IPD and PRISMA-NMA checklists (Additional file 1: Appendix Table 2).

### Methodological quality of included IPD-NMAs

**Fig. 4:**
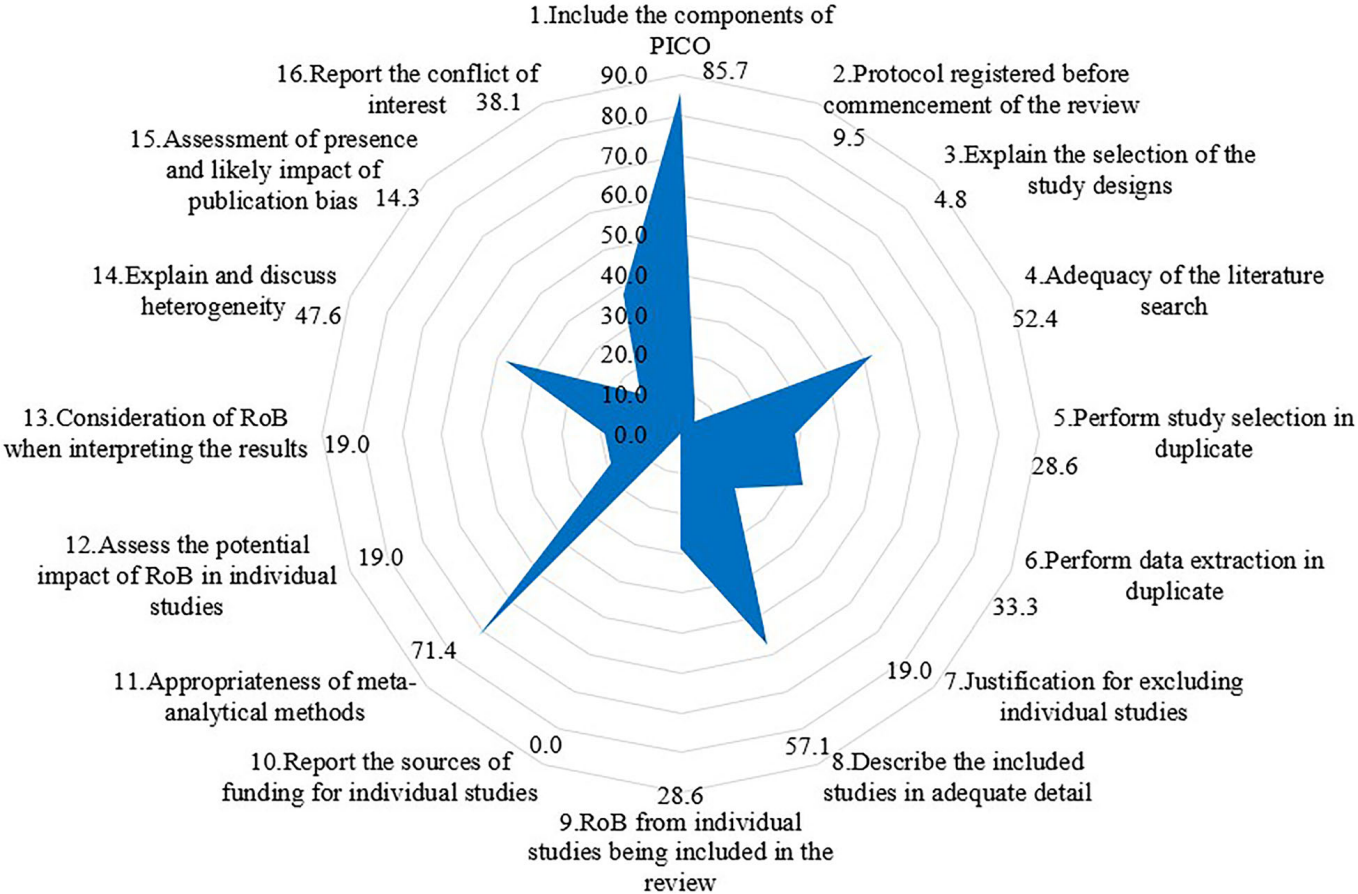
The full compliance rate of each item based on AMSTAR-2. RoB, risk of bias

Of the 21 IPD-NMAs, one was rated as high quality, one was classified as low quality, and the remaining nineteen were rated as critically low quality. Figure 4 shows the compliance rate of each AMSTAR-2 item. 85.7% of the IPD-NMAs clarified the components of PICO (population, intervention, comparison, and outcome) in the research question and inclusion criteria sections and 71.4% of the IPD-NMAs used appropriate methods (used an appropriate weighted technique, including the 1-stage or 2-stage, fixed- or random-effects model, and effect measures to combine study results, and investigated the causes of the heterogeneity) for the statistical combination of results. However, none of the IPD-NMAs reported the sources of funding for the studies included in the review, only two of the 21 IPD-NMAs pre-specified the review methods and justified the significant deviations from the protocol, and only one explained the selection of the study designs for inclusion in the review. Furthermore, only a few of the included IPD-NMAs provided a list of excluded studies, assessed the potential impact of risk of bias (RoB) in individual studies on the results of the meta-analysis, and interpreted the results considered the RoB of primary studies (Fig. 4).

Stratified analyses found no statistically significant associations between methodological quality based on the AMSTAR-2 tool and the general characteristics or statistical methods used in IPD-NMAs, including the industry funding or not, pre-specified protocol or not, Bayesian or frequentist method used, and 1-stage or 2-stage process (Additional file 1: Appendix Table 3). There were no significant improvements in the methodological quality of IPD-NMAs after the publication of the AMSTAR-2 tool (Additional file 1: Appendix Table 4).

### Result of correlation analysis

A strong positive correlation was found between the fully reported PRISMA-IPD items and fully reported AMSTAR-2 items (Pearson’s *ρ* = 0.905, *P* = 0.000) as shown in Additional file 2: Appendix Fig. 2.

## Discussion

### Findings and interpretations

This study included 21 IPD-NMAs published between 2007 and 2019, identified from a comprehensive literature search. We investigated the statistical analysis methods used and assessed the reporting quality based on PRISMA-IPD and PRISMA-NMA checklists and methodological quality using the AMSTAR-2 tool. Overall, the reporting of statistical analysis methods was suboptimal, and the reporting quality and methodological quality were low. Compliance rates were insufficient for most of the items of PRISMA-IPD, PRISMA-NMA, and AMSTAR-2.

Of the 21 IPD-NMAs, 10 combined both individual participant data and aggregate data, because IPD were not available from some relevant trials. We also found that the Bayesian IPD-NMAs were more likely to use the 1-stage process, and the Frequentist IPD-NMAs were more likely to use the 2-stage process. Reluctance to share data is a major obstacle to obtaining IPD and performing IPD meta-analysis [36, 37]. A previous study showed that IPD sharing may depend on study characteristics, including funding type, study size, study risk of bias, and treatment effect [27]. Of the 21 included IPD-NMAs, only three mentioned the retrieval bias and none assessed the impact of the retrieval bias. One of the advantages of IPD meta-analyses is that it allows the application of appropriate multiple imputation techniques to solve problems related to missing data [3]. However, only 23.8% of the IPD-NMAs reported the use of statistical techniques to handle missing participant data. Meta-analyses often have intrinsic heterogeneity, which can affect the reliability and validity of results [38]. Heterogeneity was assessed in 71.4% of the IPD-NMAs, and the sources of heterogeneity were mostly explored by performing the subgroup analysis, meta-regression analysis, or sensitivity analysis. The consistency assumption is imperative for a valid NMA, and the assessment of inconsistency should be fully reported in NMAs [24, 33]. However, no more than 45.0% of the IPD-NMAs used specific methods to evaluate consistency. The transitivity assumption is another important aspect in NMAs [39], but none of the included IPD-NMAs assessed the transitivity, which should be given more attention in the future work.

For reporting quality based on 31 PRISMA-IPD items and 5 PRISMA-NMA supplemental items, compliance rates were above 70.0% for nine items, lower than 40.0% for 14 items, and lower than 15.0% for 6 items. For IPD meta-analyses, the importance of checking and correcting any inaccuracies or errors in the IPD is self-evident [1]. However, more than 60.0% of IPD-NMAs did not explore data integrity and about 85.7% of IPD-NMAs did not report data integrity. Therefore, future IPD-NMAs should clarify which aspects of IPD were subject to data checking, how data checking was done, and report any important issues identified in checking IPD. IPD meta-analyses are particularly useful for exploring the participant-level variation of treatment response [1, 40]. Only about half of the included IPD-NMAs described methods for exploring variation in effects by the study- or participant-level characteristics and stated participant-level characteristics that were analyzed as potential effect modifiers. About 86.0% of IPD-NMAs did not present the network of geometry nor did they summarize the network geometry, which affected the understanding of NMAs [24, 41]. Furthermore, deficiencies were also identified in items related to protocol and registration, search, study selection, data collection process, risk of bias in individual studies, results of syntheses, risk of bias across studies, and funding.

Of the 16 individual AMSTAR-2 items, only four items obtained compliance rates higher than 50.0%, and seven obtained compliance rates lower than 20.0%. Only 4.8% of the IPD-NMAs explained their selection of the study designs for inclusion in the review, 19.0% provided a list of excluded studies and justified the exclusions, and none of the IPD-NMAs reported sources of funding for the studies included in the review, which is similar to the findings of other types of SRs and meta-analyses [38, 42, 43]. Therefore, these may be some common methodological shortcomings of any types of SRs. In SRs, assessing the risk of bias of primary studies and investigating publication bias are of great importance as these will gauge the validity of meta-analytic results, affect the interpretation of results, and limit our ability to draw conclusions [43, 44]. Unfortunately, only 28.6% of IPD-NMAs used a satisfactory technique for assessing the RoB in individual studies included, and only 19% of IPD-NMAs assessed the potential impact of RoB on the results of the meta-analyses. In addition, only 14.3% of the included IPD-NMAs investigated the publication bias and discussed its likely impact on the results of the review. Approximately 30.0% of the IPD-NMAs were industry-sponsored, and no more than 40.0% of IPD-NMAs reported potential conflicts of interest and funding sources, which may lead to potential risks of funding bias [21]. Publishing protocols can reduce the risk of researcher bias and outcome reporting bias [45–47]. Empirical studies have also found that a priori protocol can improve the methodological quality of SR [34, 48]. Our study showed that IPD-NMAs with a priori protocol tended to have higher methodological quality than IPD-NMAs without a priori protocol, although the difference was not statistically significant.

### Comparison of our findings with other studies

A previous study found that key methodological and reporting issues such as consistency assumption, study protocol, and statistical approaches for missing participant data were often insufficiently reported in IPD indirect comparison studies [3]. These results were similar to the findings of our analyses of IPD-NMAs. According to our knowledge, two previous studies [21, 23] explored the statistical methods, methodological and reporting characteristics of aggregate data NMAs. These two empirical studies found that, of the included NMAs, only 53.0% assessed inconsistency, 56.0% explored heterogeneity, and less than 40.0% investigated publication bias, which are also similar to the results of our study. This suggested that IPD-NMAs and aggregate data NMAs may have the same defects. Compared with Cochrane NMAs [24], IPD-NMAs had significantly lower compliance rates in 12 AMSTAR-2 items and 13 PRISMA-NMA items, revealing that both the methodological and reporting quality of IPD-NMAs were lower than Cochrane NMAs. Therefore, there was room for further improvement in both Cochrane NMAs and IPD-NMAs, such as the geometry of the network, the risk of bias in individual studies, and the assessment of inconsistency.

### Strengths and limitations

This is the first comprehensive evaluation of published IPD-NMAs, in terms of statistical methods used, reporting quality based on PRISMA-IPD and PRISMA-NMA checklists, and methodological quality based on the AMSTAR-2 tool. Furthermore, we also conducted stratified analyses to explore potential factors that may affect the reporting and methodological quality and further performed the correlation analysis to evaluate whether the reporting quality was relevant to the methodological quality. However, our study also has some limitations. First, the number of IPD-NMAs included in our study was small, although we have identified all available IPD-NMAs by conducting a comprehensive literature search. Second, our data depended on the information reported in the included IPD-NMAs, so we could not rule out the possibility that some important methods were appropriately used in the study but not reported [3]. Third, we mainly focused on the impact of selected factors on the methodological and reporting quality of IPD-NMAs. Finally, we included a very small number of IPD-NMAs and conducted a large number of statistical tests. Any significant results of stratified analyses should be interpreted with caution.

### Implications for further research and practice

Our study indicated that the reporting and methodological quality of IPD-NMAs needs to be further improved. The identified drawbacks need to be addressed, and future IPD-NMAs should be conducted according to reporting and methodological guidelines [1, 33, 35]. In this study, we assessed the reporting quality of IPD-NMAs using PRISMA-IPD and PRISMA-NMA statements. However, the PRISMA-IPD statement aims to improve the reporting quality of SRs and meta-analyses of IPD, while the PRISMA-NMA aims to improve the completeness and transparency of reporting of NMAs. Currently, there are no reporting standards aimed to enhance the reporting of IPD-NMAs. Therefore, it is necessary to develop a reporting quality tool for IPD-NMAs, incorporating relevant items from both PRISMA-IPD and PRISMA-NMA. For example, the report should be identified as a network meta-analysis of individual participant data in the title; describe methods used to explore the network geometry, transitivity assumption, and consistency assumption; and describe statistical techniques used for missing participant data and methods used to explore variation in effects.

## Conclusions

The key information on statistical methods was often missing, and compliance rates of reporting and methodological items were suboptimal in published IPD-NMAs. Methodological and reporting shortcomings include handling of missing participant data, assessment of publication bias, clarification of the IPD integrity, description of network geometry, and assessment of the consistency. Authors of future IPD-NMAs should address the identified flaws and strictly adhere to methodological and reporting guidelines. We recommend the development of a reporting quality tool that is specifically applicable to IPD-NMAs.

## Supplementary information

**Additional file 1: Appendix Word 1.** Search strategy of PubMed. **Appendix Word 2.** Result of the normality test. **Appendix Word 3.** The list of included IPD-NMAs. **Appendix Table 1.** Stratified analyses of reporting quality assessment in PRISMA-IPD items and PRISMA-NMA supplemental items. **Appendix Table 2.** Reporting quality between IPD-NMAs before and after the publication of PRISMA-IPD and PRISMA-NMA checklists. **Appendix Table 3.** Stratified analyses of methodological quality assessment in AMSTAR-2 items. **Appendix Table 4.** Methodological quality between IPD-NMAs before and after the publication of the AMSTAR-2 checklist.

**Additional file 2: Appendix Figure 1.** The full compliance rate of each PRISMA-IPD item and PRISMA-NMA supplemental item. **Appendix Figure 2.** Correlation between fully reported PRISMA-IPD items and fully reported AMSTAR-2 items.

## Data Availability

All data generated or analyzed during this study are included in this published article.

## Abbreviations

AMSTAR-2: Assessment of Multiple Systematic Reviews-2
GRADE: Grading of Recommendations Assessment, Development and Evaluation
IPD-NMAs: Individual participant data network meta-analyses
PRISMA-IPD: Preferred Reporting Items for Systematic Review and Meta-Analyses of individual participant data
PRISMA-NMA: Preferred Reporting Items for Systematic Reviews and Meta-analyses extension
RCT: Randomized controlled trial
SR: Systematic review
