# Supplementary material for: Statistical analyses and quality of individual participant data network meta-analyses were suboptimal: a cross-sectional study
Source: BMC Med. 2020 Jun 1;18:120. doi: 10.1186/s12916-020-01591-0 (PMC7262764; doi:10.1186/s12916-020-01591-0)
Supplement: Supplementary file 1 — Additional file 1: Appendix Word 1. Search strategy of PubMed. Appendix Word 2. Result of the normality test. Appendix Word 3. The list of included IPD-NMAs. Appendix Table 1. Stratified analyses of reporting quality assessment in PRISMA-IPD items and PRISMA-NMA supplemental items. Appendix Table 2. Reporting quality between IPD-NMAs before and after the publication of PRISMA-IPD and PRISMA-NMA checklists. Appendix Table 3. Stratified analyses of methodological quality assessment in AMSTAR-2 items. Appendix Table 4. Methodological quality between IPD-NMAs before and after the publication of the AMSTAR-2 checklist. [file 12916_2020_1591_MOESM1_ESM.docx]

**Statistical analyses and quality of individual participant data network meta-analyses were suboptimal: a cross-sectional study**

Ya Gao, Shuzhen Shi, Muyang Li, Xinyue Luo, Ming Liu, Kelu Yang, Junhua Zhang, Fujian Song, Jinhui Tian

| **Additional file 1** | **Page** |
| --- | --- |
| **Appendix Word 1.** Search strategy of PubMed | 2 |
| **Appendix Word 2.** Result of the normality test | 4 |
| **Appendix Word 3.** The list of included IPD-NMAs | 5 |
| **Appendix Table 1.** Stratified analyses of reporting quality assessment in PRISMA-IPD items and PRISMA-NMA supplemental items | 8 |
| **Appendix Table 2.** Reporting quality between IPD-NMAs before and after the publication of PRISMA-IPD and PRISMA-NMA checklists | 12 |
| **Appendix Table 3.** Stratified analyses of methodological quality assessment in AMSTAR-2 items | 14 |
| **Appendix Table 4.** Methodological quality between IPD-NMAs before and after the publication of the AMSTAR-2 checklist | 17 |

**Appendix Word 1.** Search strategy of PubMed

#1 Network Meta-Analysis[Mesh]

#2 network meta analysis[Title/Abstract] OR network meta analyses[Title/Abstract] OR network meta-analysis[Title/Abstract] OR network meta-analyses[Title/Abstract] OR network metaanalyses[Title/Abstract] OR network metaanalysis[Title/Abstract] OR mixed treatment comparison meta analysis[Title/Abstract] OR mixed treatment comparisons meta analyses[Title/Abstract] OR mixed treatment meta analysis[Title/Abstract] OR mixed treatment meta analyses[Title/Abstract] OR mixed treatment comparison meta-analysis[Title/Abstract] OR mixed treatment comparisons meta-analyses[Title/Abstract] OR mixed treatment meta-analysis[Title/Abstract] OR mixed treatment meta-analyses[Title/Abstract] OR mixed treatment comparison metaanalysis[Title/Abstract] OR mixed treatment comparisons metaanalyses[Title/Abstract] OR mixed treatment metaanalysis[Title/Abstract] OR mixed treatment metaanalyses[Title/Abstract] OR multiple treatment comparison meta analysis[Title/Abstract] OR multiple treatment comparisons meta analyses[Title/Abstract] OR multiple treatments meta analysis[Title/Abstract] OR multiple treatments meta analyses[Title/Abstract] OR multiple treatment meta analysis[Title/Abstract] OR multiple treatment meta analyses[Title/Abstract] OR multiple treatment comparison meta-analysis[Title/Abstract] OR multiple treatment comparisons meta-analyses[Title/Abstract] OR multiple treatments meta-analysis[Title/Abstract] OR multiple treatments meta-analyses[Title/Abstract] OR multiple treatment meta-analysis[Title/Abstract] OR multiple treatment meta-analyses[Title/Abstract] OR multiple treatment comparison metaanalysis[Title/Abstract] OR multiple treatment comparisons metaanalyses[Title/Abstract] OR multiple treatments metaanalysis[Title/Abstract] OR multiple treatments metaanalyses[Title/Abstract] OR multiple treatment metaanalysis[Title/Abstract] OR indirect comparison[Title/Abstract] OR indirect treatment[Title/Abstract] OR indirect comparisons[Title/Abstract] OR indirect treatments[Title/Abstract]

#3 #1 OR #2

#4 IPD[Title/Abstract] OR aggregate data[Title/Abstract] OR individual patient[Title/Abstract] OR individual patients[Title/Abstract] OR individual participant[Title/Abstract] OR individual participants[Title/Abstract] OR individual data[Title/Abstract] OR patient level[Title/Abstract] OR patients level[Title/Abstract] OR individual person[Title/Abstract] OR individual person[Title/Abstract]

#5 #3 AND #4

#6 maic[Title/Abstract] OR matching adjusted indirect comparison[Title/Abstract] OR matching-adjusted indirect comparison[Title/Abstract] OR matching adjusted method[Title/Abstract] OR matching-adjusted method[Title/Abstract] OR matching adjusted indirect treatment comparison[Title/Abstract] OR matching-adjusted indirect treatment comparison[Title/Abstract] OR population adjusted indirect comparison[Title/Abstract] OR population-adjusted indirect comparison[Title/Abstract] OR matched adjusted indirect comparison[Title/Abstract] OR matched-adjusted indirect comparison[Title/Abstract]

#7 #5 OR #6

**Appendix Word 2.** Result of the normality test

| **Normality test** | | | | | | |
| --- | --- | --- | --- | --- | --- | --- |
|  | Kolmogorov-Smirnov^a^ | | | Shapiro-Wilk | | |
|  | Statistic | df | Sig. | Statistic | df | Sig. |
| AMSTAR-2 | .149 | 21 | .200^*^ | .917 | 21 | .077 |
| PRISMA-IPD | .142 | 21 | .200^*^ | .949 | 21 | .323 |
| *. This is a lower bound of the true significance.  a. Lilliefors significance correction. | | | | | | |

**Appendix Word 3.** The list of included IPD-NMAs

1. Smith CT, Marson AG, Chadwick DW, Williamson PR. Multiple treatment comparisons in epilepsy monotherapy trials. Trials. 2007;8.
2. Middleton LJ, Champaneria R, Daniels JP, Bhattacharya S, Cooper KG, Hilken NH, et al. Hysterectomy, endometrial destruction, and levonorgestrel releasing intrauterine system (Mirena) for heavy menstrual bleeding: systematic review and meta-analysis of data from individual patients. British Medical Journal. 2010;341.
3. Blanchard P, Hill C, Guihenneuc-Jouyaux C, Baey C, Bourhis J, Pignon JP, et al. Mixed treatment comparison meta-analysis of altered fractionated radiotherapy and chemotherapy in head and neck cancer. Journal of Clinical Epidemiology. 2011;64:985-92.
4. Cope S, Capkun-Niggli G, Gale R, Lassen C, Owen R, Ouwens M, et al. Efficacy of Once-Daily Indacaterol Relative to Alternative Bronchodilators in COPD: A Patient-Level Mixed Treatment Comparison. Value in Health. 2012;15:524-33.
5. Cope S, Zhang J, Williams J, Jansen JP. Efficacy of once-daily indacaterol 75 mu g relative to alternative bronchodilators in COPD: A study level and a patient level network meta-analysis. Bmc Pulmonary Medicine. 2012;12.
6. Daniels JP, Middleton LJ, Champaneria R, Khan KS, Cooper K, Mol BW, et al. Second generation endometrial ablation techniques for heavy menstrual bleeding: network meta-analysis. BMJ (Clinical research ed). 2012;344:e2564.
7. Szegedi A, Verweij P, van Duijnhoven W, Mackle M, Cazorla P, Fennema H. Meta-analyses of the efficacy of asenapine for acute schizophrenia: comparisons with placebo and other antipsychotics. The Journal of clinical psychiatry. 2012;73:1533-40.
8. Whegang Youdom S, Samson A, Basco LK, Thalabard JC. Multiple treatment comparisons in a series of anti-malarial trials with an ordinal primary outcome and repeated treatment evaluations. Malaria journal. 2012;11:147.
9. Bhala N, Emberson J, Merhi A, Abramson S, Arber N, Baron JA, et al. Vascular and upper gastrointestinal effects of non-steroidal anti-inflammatory drugs: meta-analyses of individual participant data from randomised trials. Lancet (London, England). 2013;382:769-79.
10. Ellis AG, Reginster JY, Luo X, Cappelleri JC, Chines A, Sutradhar S, et al. Bazedoxifene versus oral bisphosphonates for the prevention of nonvertebral fractures in postmenopausal women with osteoporosis at higher risk of fracture: A network meta-analysis. Value in Health. 2014;17:424-32.
11. Ellis AG, Reginster JY, Luo XM, Bushmakin AG, Williams R, Sutradhar S, et al. Indirect comparison of bazedoxifene vs oral bisphosphonates for the prevention of vertebral fractures in postmenopausal osteoporotic women. Current Medical Research and Opinion. 2014;30:1617-26.
12. Goodacre S, Stevens JW, Pandor A, Poku E, Ren SJ, Cantrell A, et al. Prehospital Noninvasive Ventilation for Acute Respiratory Failure: Systematic Review, Network Meta-analysis, and Individual Patient Data Meta-analysis. Academic Emergency Medicine. 2014;21:960-70.
13. Mills EJ, Lester R, Thorlund K, Lorenzi M, Muldoon K, Kanters S, et al. Interventions to promote adherence to antiretroviral therapy in Africa: A network meta-analysis. The Lancet HIV. 2014;1:e104-e11.
14. Palmerini T, Sangiorgi D, Valgimigli M, Biondi-Zoccai G, Feres F, Abizaid A, et al. Short- Versus Long-Term Dual Antiplatelet Therapy After Drug-Eluting Stent Implantation An Individual Patient Data Pairwise and Network Meta-Analysis. Journal of the American College of Cardiology. 2015;65:1092-102.
15. Woods B, Hawkins N, Mealing S, Sutton A, Abraham WT, Beshai JF, et al. Individual patient data network meta-analysis of mortality effects of implantable cardiac devices. Heart. 2015;101:1800-6.
16. Nevitt SJ, Sudell M, Weston J, Smith CT, Marson AG. Antiepileptic drug monotherapy for epilepsy: a network meta-analysis of individual participant data. Cochrane Database of Systematic Reviews. 2017;6:CD011412.
17. Palmerini T, Della Riva D, Benedetto U, Bacchi Reggiani L, Feres F, Abizaid A, et al. Three, six, or twelve months of dual antiplatelet therapy after DES implantation in patients with or without acute coronary syndromes: an individual patient data pairwise and network meta-analysis of six randomized trials and 11 473 patients. Eur Heart J. 2017;38:1034-43.
18. Ribassin-Majed L, Marguet S, Lee AWM, Ng WT, Ma J, Chan ATC, et al. What is the best treatment of locally advanced nasopharyngeal carcinoma? an individual patient data network meta-analysis. Journal of Clinical Oncology. 2017;35:498-505.
19. Muston D, Korobelnik JF, Reason T, Hawkins N, Chatzitheofilou I, Ryan F, et al. An efficacy comparison of anti-vascular growth factor agents and laser photocoagulation in diabetic macular edema: a network meta-analysis incorporating individual patient-level data. BMC ophthalmology. 2018;18:340.
20. Spertus J, Horvitz-Lennon M, Abing H, Normand SL. Risk of weight gain for specific antipsychotic drugs: A meta-analysis. npj Schizophrenia. 2018;4.
21. Petit C, Blanchard P, Pignon J, Lueza B. Individual patient data network meta-analysis using either restricted mean survival time difference or hazard ratios: Is there a difference? A case study on locoregionally advanced nasopharyngeal carcinomas. Systematic Reviews. 2019;8.

**Appendix Table 1.** Stratified analyses of reporting quality assessment in PRISMA-IPD items and PRISMA-NMA supplemental items

| Section | Items | With statistician or epidemiologist vs. Without statistician or epidemiologist | | | With a priori protocol vs. Without a priori protocol | | | Non-industry funding vs. Industry funding* | | | IPD-NMAs with Bayesian method vs. IPD-NMAs with Frequentist method | | | IPD-NMAs with 1-stage process vs. IPD-NMAs with 2-stage process | | |
| --- | --- | --- | --- | --- | --- | --- | --- | --- | --- | --- | --- | --- | --- | --- | --- | --- |
| PRISMA-IPD items |  | With  (n=7) | Without  (n=14) | *P*-value | With  (n=6) | Without  (n=15) | *P*-value | Non-industry  (n=14) | Industry  (n=9) | *P*-value | Bayesian  (n=12) | Frequentist  (n=9) | *P*-value | 1-stage  (n=14) | 2-stage  (n=7) | *P*-value |
| Title/Abstract | 1. Title | 3(42.9) | 9(64.3) | 0.397 | 4(66.7) | 8(53.3) | 0.659 | 9(64.3) | 5(55.6) | 1.000 | 6(50.0) | 6(66.7) | 0.660 | 7(50.0) | 5(71.4) | 0.642 |
|  | 2. Structured summary | 6(85.7) | 14(100.0) | 0.333 | 6(100.0) | 14(93.3) | 1.000 | 13(92.9) | 9(100.0) | 1.000 | 11(91.7) | 9(100.0) | 1.000 | 13(92.9) | 7(100.0) | 1.000 |
| Introduction | 3. Rationale | 7(100.0) | 14(100.0) | —— | 6(100.0) | 15(100.0) | —— | 14(100.0) | 9(100.0) | —— | 12(100.0) | 9(100.0) | —— | 14 (100.0) | 7 (100.0) | —— |
|  | 4. Objectives | 5(71.4) | 11(78.6) | 1.000 | 5(83.3) | 11(73.3) | 1.000 | 11 (78.6) | 6 (66.7) | 0.643 | 9 (75.0) | 7 (77.8) | 1.000 | 11(78.6) | 5(71.4) | 1.000 |
| Methods | 5. Protocol and registration | 0(0.0) | 2(14.3) | 0.533 | 2(33.3) | 0(0.0) | 0.071 | 2(14.3) | 0(0.0) | 0.502 | 1(8.3) | 1(11.1) | 1.000 | 2(14.3) | 0(0.0) | 0.533 |
|  | 6. Eligibility criteria | 4(57.1) | 10(71.4) | 0.638 | 4(66.7) | 10(66.7) | 1.000 | 10(71.4) | 6(66.7) | 1.000 | 8(66.7) | 6(66.7) | 1.000 | 10(71.4) | 4(57.1) | 0.638 |
|  | 7. Information sources | 4(57.1) | 10(71.4) | 0.638 | 6(100.0) | 8(53.3) | 0.061 | 10(71.4) | 5(55.6) | 0.657 | 7(58.3) | 7(77.8) | 0.642 | 8(57.1) | 6(85.7) | 0.337 |
|  | 8. Search | 2(28.6) | 7(50.0) | 0.642 | 5(83.3) | 4(26.7) | 0.046 | 6(42.9) | 3(33.3) | 1.000 | 5(41.7) | 4(44.4) | 1.000 | 6(42.9) | 3(42.9) | 1.000 |
|  | 9. Study selection | 0(0.0) | 5(35.7) | 0.123 | 3(50.0) | 2(13.3) | 0.115 | 3(21.4) | 2(22.2) | 1.000 | 4(33.3) | 1(11.1) | 0.338 | 5(35.7) | 0(0.0) | 0.123 |
|  | 10. Data collection process | 2(28.6) | 4(28.6) | 1.000 | 4(66.7) | 2(13.3) | 0.031 | 6(42.9) | 1(11.1) | 0.176 | 2(16.7) | 4(44.4) | 0.331 | 3(21.4) | 3(42.9) | 0.354 |
|  | 11. Data items | 2(28.6) | 8(57.1) | 0.361 | 3(50.0) | 7(46.7) | 1.000 | 6(42.9) | 5(55.6) | 0.680 | 8(66.7) | 2(22.2) | 0.080 | 10(71.4) | 0(0.0) | 0.004 |
|  | A1. IPD integrity | 1(14.3) | 7(50.0) | 0.174 | 2(33.3) | 6(40.0) | 1.000 | 6(42.9) | 4(44.4) | 1.000 | 5(41.7) | 3(33.3) | 1.000 | 6(42.9) | 2(28.6) | 0.656 |
|  | 12. Risk of bias in individual studies | 0(0.0) | 6(42.9) | 0.061 | 3(50.0) | 3(20.0) | 0.291 | 5(35.7) | 2(22.2) | 0.657 | 3(25.0) | 3(33.3) | 1.000 | 5(35.7) | 1(14.3) | 0.613 |
|  | 13. Specification of outcomes and effect measures | 5(71.4) | 9(64.3) | 1.000 | 5(83.3) | 9(60.0) | 0.613 | 10(71.4) | 4(44.4) | 0.383 | 6(50.0) | 8(88.9) | 0.159 | 8(57.1) | 6(85.7) | 0.337 |
|  | 14. Synthesis methods | 6(85.7) | 11(78.6) | 1.000 | 5(83.3) | 12(80.0) | 1.000 | 13(92.9) | 7(77.8) | 0.538 | 9(75.0) | 8(88.9) | 0.603 | 11(78.6) | 6(85.7) | 1.000 |
|  | A2. Exploration of variation in effects | 2(28.6) | 9(64.3) | 0.183 | 3(50.0) | 8(53.3) | 1.000 | 7(50.0) | 6(66.7) | 0.669 | 7(58.3) | 4(44.4) | 0.670 | 7(50.0) | 4(57.1) | 1.000 |
|  | 15. Risk of bias across studies | 0(0.0) | 2(14.3) | 0.533 | 1(16.7) | 1(6.7) | 0.500 | 2(14.3) | 1(11.1) | 1.000 | 1(8.3) | 1(11.1) | 1.000 | 1(7.1) | 1(14.3) | 1.000 |
|  | 16. Additional analyses | 5(71.4) | 9(64.3) | 1.000 | 4(66.7) | 10(66.7) | 1.000 | 10(71.4) | 6(66.7) | 1.000 | 9(75.0) | 5(55.6) | 0.397 | 9(64.3) | 5(71.4) | 1.000 |
| Results | 17. Study selection and IPD obtained | 2(28.6) | 10(71.4) | 0.159 | 5(83.3) | 7(46.7) | 0.178 | 7(50.0) | 5(55.6) | 1.000 | 7(58.3) | 5(55.6) | 1.000 | 9(64.3) | 3(42.9) | 0.397 |
|  | 18. Study characteristics | 1(14.3) | 9(64.3) | 0.063 | 5(83.3) | 5(33.3) | 0.063 | 5(35.7) | 4(44.4) | 1.000 | 6(50.0) | 4(44.4) | 1.000 | 8(57.1) | 2(28.6) | 0.361 |
|  | A3. IPD integrity | 1(14.3) | 2(14.3) | 1.000 | 1(16.7) | 2(13.3) | 1.000 | 3(21.4) | 1(11.1) | 1.000 | 1(8.3) | 2(22.2) | 0.553 | 2(14.3) | 1(14.3) | 1.000 |
|  | 19. Risk of bias within studies | 0(0.0) | 5(35.7) | 0.123 | 3(50.0) | 2(13.3) | 0.115 | 5(35.7) | 1(11.1) | 0.340 | 2(16.7) | 3(33.3) | 0.611 | 4(28.6) | 1(14.3) | 0.624 |
|  | 20. Results of individual studies | 5(71.4) | 11(78.6) | 1.000 | 5(83.3) | 11(73.3) | 1.000 | 12(85.7) | 6(66.7) | 0.343 | 9(75.0) | 7(77.8) | 1.000 | 11(78.6) | 5(71.4) | 1.000 |
|  | 21. Results of syntheses | 2(28.6) | 5(35.7) | 1.000 | 5(83.3) | 2(13.3) | 0.006 | 6(42.9) | 0(0.0) | 0.048 | 3(25.0) | 4(44.4) | 0.397 | 4(28.6) | 3(42.9) | 0.638 |
|  | 22. Risk of bias across studies | 0(0.0) | 3(21.4) | 0.521 | 2(33.3) | 1(6.7) | 0.184 | 3(21.4) | 1(11.1) | 1.000 | 2(16.7) | 1(11.1) | 1.000 | 2(14.3) | 1(14.3) | 1.000 |
|  | 23. Additional analyses | 7(100.0) | 13(92.9) | 1.000 | 6(100.0) | 14(93.3) | 1.000 | 14(100.0) | 8(88.9) | 0.391 | 12(100.0) | 8(88.9) | 0.429 | 14(100.0) | 6(85.7) | 0.333 |
| Discussion | 24. Summary of evidence | 7(100.0) | 13(92.9) | 1.000 | 5(83.3) | 15(100.0) | 0.286 | 13(92.9) | 9(100.0) | 1.000 | 11(91.7) | 9(100.0) | 1.000 | 13(92.9) | 7(100.0) | 1.000 |
|  | 25. Strengths and limitations | 4(57.1) | 7(50.0) | 1.000 | 3(50.0) | 8(53.3) | 1.000 | 6(42.9) | 5(55.6) | 0.680 | 6(50.0) | 5(55.6) | 1.000 | 8(57.1) | 3(42.9) | 0.659 |
|  | 26. Conclusions | 7(100.0) | 14(100.0) | —— | 6(100.0) | 15(100.0) | —— | 14(100.0) | 9(100.0) | —— | 12(100.0) | 9(100.0) | —— | 14(100.0) | 7(100.0) | —— |
|  | A4. Implications | 6(85.7) | 11(78.6) | 1.000 | 5(83.3) | 12(80.0) | 1.000 | 12(85.7) | 6(66.7) | 0.343 | 10(83.3) | 7(77.8) | 1.000 | 12(85.7) | 5(71.4) | 0.574 |
| Funding | 27. Funding | 3(42.9) | 5(35.7) | 1.000 | 4(66.7) | 4(26.7) | 0.146 | 6(42.9) | 2(22.2) | 0.400 | 5(41.7) | 3(33.3) | 1.000 | 5(35.7) | 3(42.9) | 1.000 |
| PRISMA-NMA supplemental items | S1. Geometry of the network | 0(0.0) | 3(21.4) | 0.521 | 1(16.7) | 2(13.3) | 1.000 | 3(21.4) | 1(11.1) | 1.000 | 2(16.7) | 1(11.1) | 1.000 | 3(21.4) | 0(0.0) | 0.521 |
|  | S2. Assessment of inconsistency | 5(71.4) | 4(28.6) | 0.159 | 4(66.7) | 5(33.3) | 0.331 | 6(42.9) | 3(33.3) | 1.000 | 4(33.3) | 5(55.6) | 0.396 | 6(42.9) | 3(42.9) | 1.000 |
|  | S3. Presentation of network structure | 3(42.9) | 9(64.3) | 0.397 | 4(66.7) | 8(53.3) | 0.659 | 8(57.1) | 5(55.6) | 1.000 | 7(58.3) | 5(55.6) | 1.000 | 8(57.1) | 4(57.1) | 1.000 |
|  | S4. Summary of network geometry | 1(14.3) | 2(14.3) | 1.000 | 1(16.7) | 2(13.3) | 1.000 | 2(14.3) | 1(11.1) | 1.000 | 1(8.3) | 2(22.2) | 0.553 | 2(14.3) | 1(14.3) | 1.000 |
|  | S5. Exploration for inconsistency | 5(71.4) | 2(14.3) | 0.017 | 4(66.7) | 3(20.0) | 0.120 | 6(42.9) | 1(11.1) | 0.176 | 2(16.7) | 5(55.6) | 0.159 | 4(28.6) | 3(42.9) | 0.638 |

*3 IPD-NMAs were supported by both industry and non-industry funding.

**Appendix Table 2.** Reporting quality between IPD-NMAs before and after the publication of PRISMA-IPD and PRISMA-NMA checklists.

| Section | Items | ≤2015(n=15) | >2015(n=6) |
| --- | --- | --- | --- |
| PRISMA-IPD items |  |  |  |
| Title/Abstract | 1. Title | 7(46.7) | 5(83.3) |
|  | 2. Structured summary | 15(100.0) | 5(83.3) |
| Introduction | 3. Rationale | 15(100.0) | 6(100.0) |
|  | 4. Objectives | 12(80.0) | 4(66.7) |
| Methods | 5. Protocol and registration | 1(6.7) | 1(16.7) |
|  | 6. Eligibility criteria | 10(66.7) | 4(66.7) |
|  | 7. Information sources | 9(60.0) | 5(83.3) |
|  | 8. Search | 7(46.7) | 2(33.3) |
|  | 9. Study selection | 4(26.7) | 1(16.7) |
|  | 10. Data collection process | 5(33.3) | 1(16.7) |
|  | 11. Data items | 8(53.3) | 2(33.3) |
|  | A1. IPD integrity | 6(40.0) | 2(33.3) |
|  | 12. Risk of bias in individual studies | 4(26.7) | 2(33.3) |
|  | 13. Specification of outcomes and effect measures | 8(53.3) | 6(100.0) |
|  | 14. Synthesis methods | 12(80.0) | 5(83.3) |
|  | A2. Exploration of variation in effects | 8(53.3) | 3(50.0) |
|  | 15. Risk of bias across studies | 1(6.7) | 1(16.7) |
|  | 16. Additional analyses | 10(66.7) | 4(66.7) |
| Results | 17. Study selection and IPD obtained | 9(60.0) | 3(50.0) |
|  | 18. Study characteristics | 7(46.7) | 3(50.0) |
|  | A3. IPD integrity | 2(13.3) | 1(16.7) |
|  | 19. Risk of bias within studies | 3(20.0) | 2(33.3) |
|  | 20. Results of individual studies | 11(73.3) | 5(83.3) |
|  | 21. Results of syntheses | 6(40.0) | 1(16.7) |
|  | 22. Risk of bias across studies | 2(13.3) | 1(16.7) |
|  | 23. Additional analyses | 14(93.3) | 6(100.0) |
| Discussion | 24. Summary of evidence | 14(93.3) | 6(100.0) |
|  | 25. Strengths and limitations | 8(53.3) | 3(50.0) |
|  | 26. Conclusions | 15(100.0) | 6(100.0) |
|  | A4. Implications | 12(80.0) | 5(83.3) |
| Funding | 27. Funding | 5(33.3) | 3(50.0) |
| PRISMA-NMA supplemental items | S1. Geometry of the network | 2(13.3) | 1(16.7) |
|  | S2. Assessment of inconsistency | 6(40.0) | 3(50.0) |
|  | S3. Presentation of network structure | 7(46.7) | 5(83.3) |
|  | S4. Summary of network geometry | 0(0.0) | 3(50.0) |
|  | S5. Exploration for inconsistency | 4(26.7) | 3(50.0) |
|  | Total items (mean±sd) | 17.93±4.73 | 19.83±8.42 |

**Appendix Table 3.** Stratified analyses of methodological quality assessment in AMSTAR-2 items.

| Items | With statistician or epidemiologist vs. Without statistician or epidemiologist | | | With a priori protocol vs. Without a priori protocol | | | Non-industry funding vs. Industry funding* | | | IPD-NMAs with Bayesian method vs. IPD-NMAs with Frequentist method | | | IPD-NMAs with 1-stage process vs. IPD-NMAs with 2-stage process | | |
| --- | --- | --- | --- | --- | --- | --- | --- | --- | --- | --- | --- | --- | --- | --- | --- |
|  | With  (n=7) | Without  (n=14) | *P*-value | With  (n=6) | Without  (n=15) | *P*-value | Non-industry  (n=14) | Industry  (n=9) | *P*-value | Bayesian  (n=12) | Frequentist  (n=9) | *P*-value | 1-stage  (n=14) | 2-stage  (n=7) | *P*-value |
| 1. Did the research questions and inclusion criteria for the review include the components of PICO? | 4(57.1) | 14(100.0) | 0.026 | 5(83.3) | 13(86.7) | 1.000 | 11(78.6) | 8(88.9) | 1.000 | 11(91.7) | 7(77.8) | 0.553 | 13(92.9) | 5(71.4) | 0.247 |
| 2. Did the report of the review contain an explicit statement that the review methods were established prior to the conduct of the review and did the report justify any significant deviations from the protocol? | 0(0.0) | 2(14.3) | 0.533 | 2(33.3) | 0(0.0) | 0.071 | 2(14.3) | 0(0.0) | 0.502 | 1(8.3) | 1(11.1) | 1.000 | 2(14.3) | 0(0.0) | 0.533 |
| 3. Did the review authors explain their selection of the study designs for inclusion in the review? | 0(0.0) | 1(7.1) | 1.000 | 1(16.7) | 0(0.0) | 0.286 | 1(7.1) | 0(0.0) | 1.000 | 0(0.0) | 1(11.1) | 0.429 | 1(7.1) | 0(0.0) | 1.000 |
| 4. Did the review authors use a comprehensive literature search strategy? | 2(28.6) | 9(64.3) | 0.183 | 4(66.7) | 7(46.7) | 0.635 | 8(57.1) | 5(55.6) | 1.000 | 6(50.0) | 5(55.6) | 1.000 | 7(50.0) | 4(57.1) | 1.000 |
| 5. Did the review authors perform study selection in duplicate? | 1(14.3) | 5(35.7) | 0.613 | 3(50.0) | 3(20.0) | 0.291 | 4(28.6) | 2(22.2) | 1.000 | 4(33.3) | 2(22.2) | 0.659 | 6(42.9) | 0(0.0) | 0.061 |
| 6. Did the review authors perform data extraction in duplicate? | 3(42.9) | 4(28.6) | 0.638 | 4(66.7) | 3(20.0) | 0.120 | 7(50.0) | 1(11.1) | 0.086 | 2(16.7) | 5(55.6) | 0.159 | 4(28.6) | 3(42.9) | 0.638 |
| 7. Did the review authors provide a list of excluded studies and justify the exclusions? | 0(0.0) | 4(28.6) | 0.255 | 2(33.3) | 2(13.3) | 0.544 | 2(14.3) | 2(22.2) | 1.000 | 3(25.0) | 1(11.1) | 0.603 | 4(28.6) | 0(0.0) | 0.255 |
| 8. Did the review authors describe the included studies in adequate detail? | 2(28.6) | 10(71.4) | 0.159 | 5(83.3) | 7(46.7) | 0.178 | 8(57.1) | 4(44.4) | 0.680 | 7(58.3) | 5(55.6) | 1.000 | 9(64.3) | 3(42.9) | 0.397 |
| 9. Did the review authors use a satisfactory technique for assessing the risk of bias (RoB) in individual studies that were included in the review? | 0(0.0) | 6(42.9) | 0.061 | 3(50.0) | 3(20.0) | 0.291 | 5(35.7) | 2(22.2) | 0.657 | 3(25.0) | 3(33.3) | 1.000 | 5(35.7) | 1(14.3) | 0.613 |
| 10. Did the review authors report on the sources of funding for the studies included in the review? | 0(0.0) | 0(0.0) | —— | 0(0.0) | 0(0.0) | —— | 0(0.0) | 0(0.0) | —— | 0(0.0) | 0(0.0) | —— | 0(0.0) | 0(0.0) | —— |
| 11. If meta-analysis was performed, did the review authors use appropriate methods for statistical combination of results? | 5(71.4) | 10(71.4) | 1.000 | 6(100.0) | 9(60.0) | 0.123 | 12(85.7) | 4(44.4) | 0.066 | 7(58.3) | 8(88.9) | 0.178 | 9(64.3) | 6(85.7) | 0.613 |
| 12. If meta-analysis was performed, did the review authors assess the potential impact of RoB in individual studies on the results of the meta-analysis or other evidence synthesis? | 1(14.3) | 3(21.4) | 1.000 | 2(33.3) | 2(13.3) | 0.544 | 3(21.4) | 1(11.1) | 1.000 | 2(16.7) | 2(22.2) | 1.000 | 3(21.4) | 1(14.3) | 1.000 |
| 13. Did the review authors account for RoB in primary studies when interpreting/discussing the results of the review? | 1(14.3) | 3(21.4) | 1.000 | 2(33.3) | 2(13.3) | 0.544 | 3(21.4) | 1(11.1) | 1.000 | 2(16.7) | 2(22.2) | 1.000 | 3(21.4) | 1(14.3) | 1.000 |
| 14. Did the review authors provide a satisfactory explanation for, and discussion of, any heterogeneity observed in the results of the review? | 4(57.1) | 6(42.9) | 0.659 | 3(50.0) | 7(46.7) | 1.000 | 7(50.0) | 3(33.3) | 0.669 | 5(41.7) | 5(55.6) | 0.670 | 7(50.0) | 3(42.9) | 1.000 |
| 15. If they performed quantitative synthesis did the review authors carry out an adequate investigation of publication bias (small study bias) and discuss its likely impact on the results of the review? | 0(0.0) | 3(21.4) | 0.521 | 2(33.3) | 1(6.7) | 0.184 | 3(21.4) | 1(11.1) | 1.000 | 2(16.7) | 1(11.1) | 1.000 | 2(14.3) | 1(14.3) | 1.000 |
| 16. Did the review authors report any potential sources of conflict of interest, including any funding they received for conducting the review? | 3(42.9) | 5(35.7) | 1.000 | 4(66.7) | 4(26.7) | 0.146 | 6(42.9) | 2(22.2) | 0.400 | 5(41.7) | 3(33.3) | 1.000 | 5(35.7) | 3(42.9) | 1.000 |

*3 IPD-NMAs were supported by both industry and non-industry funding.

**Appendix Table 4.** Methodological quality between IPD-NMAs before and after the publication of the AMSTAR-2 checklist.

| Items | ≤2017(n=18) | >2017(n=3) |
| --- | --- | --- |
| 1. Did the research questions and inclusion criteria for the review include the components of PICO? | 16(88.9) | 2(66.7) |
| 2. Did the report of the review contain an explicit statement that the review methods were established prior to the conduct of the review and did the report justify any significant deviations from the protocol? | 2(11.1) | 0(0.0) |
| 3. Did the review authors explain their selection of the study designs for inclusion in the review? | 1(5.6) | 0(0.0) |
| 4. Did the review authors use a comprehensive literature search strategy? | 10(55.6) | 1(33.3) |
| 5. Did the review authors perform study selection in duplicate? | 6(33.3) | 0(0.0) |
| 6. Did the review authors perform data extraction in duplicate? | 7(38.9) | 0(0.0) |
| 7. Did the review authors provide a list of excluded studies and justify the exclusions? | 4(22.2) | 0(0.0) |
| 8. Did the review authors describe the included studies in adequate detail? | 12(66.7) | 0(0.0) |
| 9. Did the review authors use a satisfactory technique for assessing the risk of bias (RoB) in individual studies that were included in the review? | 6(33.3) | 0(0.0) |
| 10. Did the review authors report on the sources of funding for the studies included in the review? | 0(0.0) | 0(0.0) |
| 11. If meta-analysis was performed, did the review authors use appropriate methods for statistical combination of results? | 13(72.2) | 2(66.7) |
| 12. If meta-analysis was performed, did the review authors assess the potential impact of RoB in individual studies on the results of the meta-analysis or other evidence synthesis? | 4(22.2) | 0(0.0) |
| 13. Did the review authors account for RoB in primary studies when interpreting/discussing the results of the review? | 4(22.2) | 0(0.0) |
| 14. Did the review authors provide a satisfactory explanation for, and discussion of, any heterogeneity observed in the results of the review? | 9(50.0) | 1(33.3) |
| 15. If they performed quantitative synthesis did the review authors carry out an adequate investigation of publication bias (small study bias) and discuss its likely impact on the results of the review? | 3(16.7) | 0(0.0) |
| 16. Did the review authors report any potential sources of conflict of interest, including any funding they received for conducting the review? | 6(33.3) | 2(66.7) |
| Total items (mean±sd) | 5.72±3.83 | 2.67±2.08 |
