# Supplementary material for: Statistical analyses and quality of individual participant data network meta-analyses were suboptimal: a cross-sectional study
Source: BMC Med. 2020 Jun 1;18:120. doi: 10.1186/s12916-020-01591-0 (PMC7262764; doi:10.1186/s12916-020-01591-0)

**Statistical analyses and quality of individual participant data network meta-analyses were suboptimal: a cross-sectional study**

Ya Gao, Shuzhen Shi, Muyang Li, Xinyue Luo, Ming Liu, Kelu Yang, Junhua Zhang, Fujian Song, Jinhui Tian

| **Additional file 2** | **Page** |
| --- | --- |
| **Appendix Figure 1.** The full compliance rate of each PRISMA-IPD item and PRISMA-NMA supplemental item. | 2 |
| **Appendix Figure 2.** Correlation between fully reported PRISMA-IPD items and fully reported AMSTAR-2 items. | 3 |

**Appendix Figure 1.** The full compliance rate of each PRISMA-IPD item and PRISMA-NMA supplemental item.


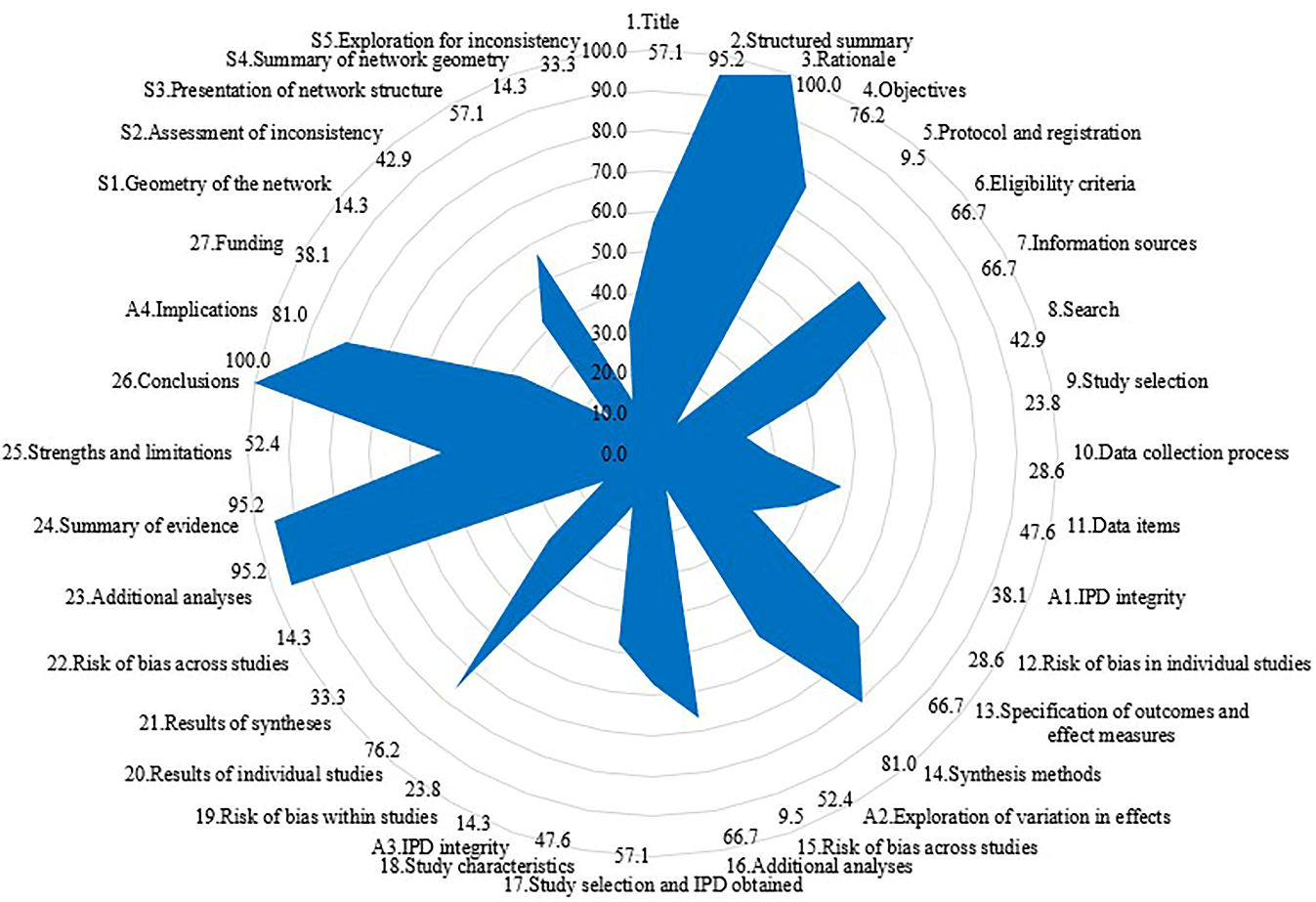


**Appendix Figure 2.** Correlation between fully reported PRISMA-IPD items and fully reported AMSTAR-2 items.


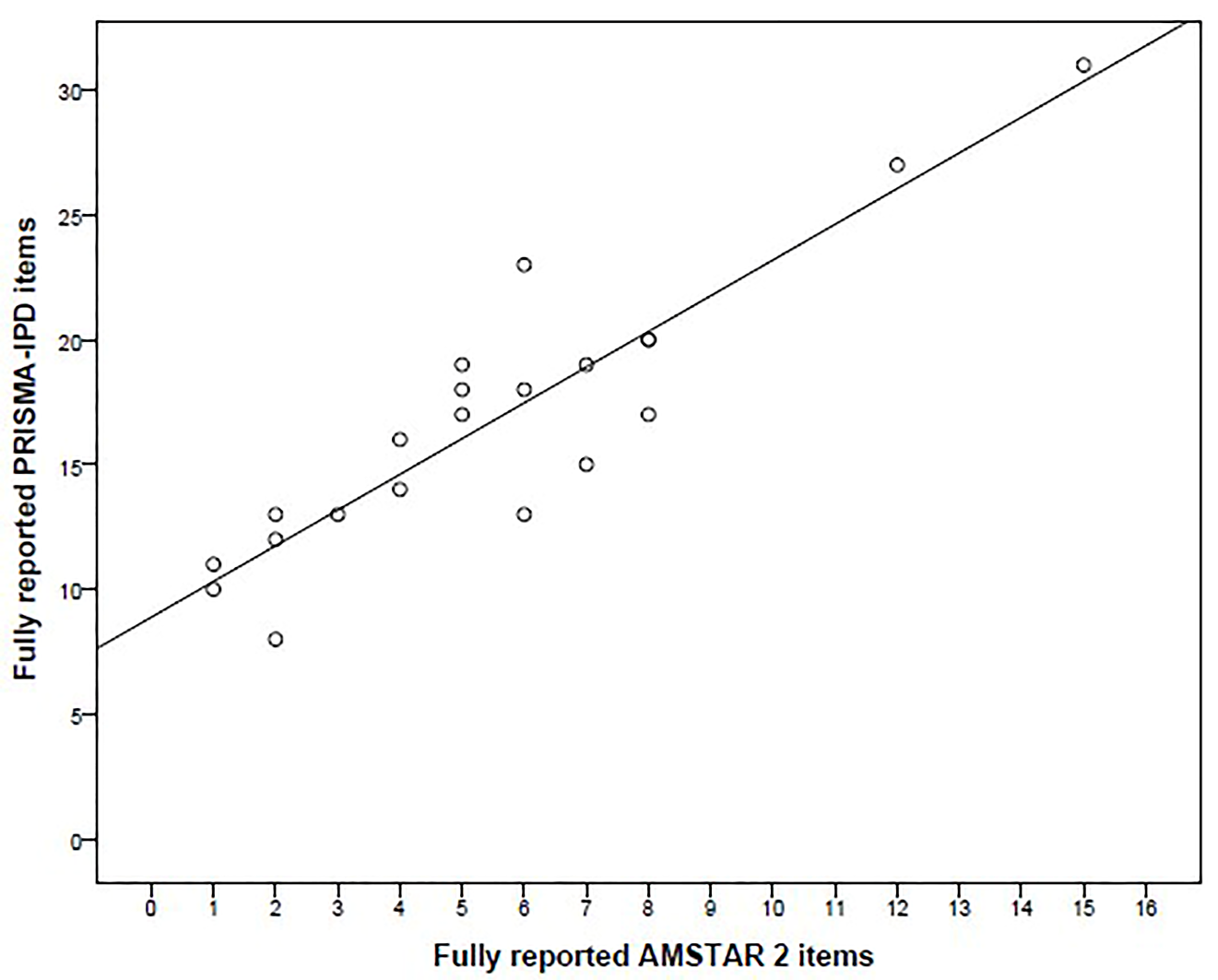

Supplement: Supplementary file 2 — Additional file 2: Appendix Figure 1. The full compliance rate of each PRISMA-IPD item and PRISMA-NMA supplemental item. Appendix Figure 2. Correlation between fully reported PRISMA-IPD items and fully reported AMSTAR-2 items. [file 12916_2020_1591_MOESM2_ESM.docx]
